# First Experience and Prospective Evaluation on Feasibility and Acute Toxicity of Online Adaptive Radiotherapy of the Prostate Bed as Salvage Treatment in Patients with Biochemically Recurrent Prostate Cancer on a 1.5T MR-Linac

**DOI:** 10.3390/jcm11164651

**Published:** 2022-08-09

**Authors:** Daniel Wegener, Alexandra Thome, Frank Paulsen, Cihan Gani, Jessica Boldt, Sarah Butzer, Daniela Thorwarth, David Moennich, Marcel Nachbar, Arndt-Christian Müller, Daniel Zips, Simon Boeke

**Affiliations:** 1 Department of Radiation Oncology, Eberhard Karls University, 72076 Tuebingen, Germany; 2 Section for Biomedical Physics, Department of Radiation Oncology, Eberhard Karls University, 72076 Tuebingen, Germany; 3 German Cancer Consortium (DKTK), Partner Site Tübingen and German Cancer Research Center (DKFZ), 69120 Heidelberg, Germany; 4 Department of Radiation Oncology, Klinikum Ludwigsburg, 71640 Ludwigsburg, Germany; 5 Department of Radiation Oncology, Charité Berlin, 10117 Berlin, Germany

**Keywords:** MR-Linac, MR-guided RT, postoperative RT of prostate cancer, adaptive RT

## Abstract

Introduction: Novel MRI-linear accelerator hybrids (MR-Linacs, MRL) promise an optimization of radiotherapy (RT) through daily MRI imaging with enhanced soft tissue contrast and plan adaptation on the anatomy of the day. These features might potentially improve salvage RT of prostate cancer (SRT), where the clinical target volume is confined by the mobile organs at risk (OAR) rectum and bladder. So far, no data exist about the feasibility of the MRL technology for SRT. In this study, we prospectively examined patients treated with SRT on a 1.5 T MRL and report on workflow, feasibility and acute toxicity. Patients and Methods: Sixteen patients were prospectively enrolled within the MRL-01 study (NCT: NCT04172753). All patients were staged and had an indication for SRT after radical prostatectomy according to national guidelines. RT consisted of 66 Gy in 33 fractions or 66.5/70 Gy in 35 fractions in case of a defined high-risk region. On the 1.5 T MRL, daily plan adaption was performed using one of two workflows: adapt to shape (ATS, using contour adaptation and replanning) or adapt to position (ATP, rigid replanning onto the online anatomy with virtual couch shift). Duration of treatment steps, choice of workflow and treatment failure were recorded for each fraction of each patient. Patient-reported questionnaires about patient comfort were evaluated as well as extensive reporting of acute toxicity (patient reported and clinician scored). Results: A total of 524/554 (94.6%) of fractions were successfully treated on the MRL. No patient-sided treatment failures occurred. In total, ATP was chosen in 45.7% and ATS in 54.3% of fractions. In eight cases, ATP was performed on top of the initial ATS workflow. Mean (range) duration of all fractions (on-table time until end of treatment) was 25.1 (17.6–44.8) minutes. Mean duration of the ATP workflow was 20.60 (17.6–25.2) minutes and of the ATS workflow 31.3 (28.2–34.1) minutes. Patient-reported treatment experience questionnaires revealed high rates of tolerability of the treatment procedure. Acute toxicity (RTOG, CTC as well as patient-reported CTC, IPSS and ICIQ) during RT and 3 months after was mild to moderate with a tendency of recovery to baseline levels at 3 months post RT. No G3+ toxicity was scored for any item. Conclusions: In this first report on SRT of prostate cancer patients on a 1.5 T MRL, we could demonstrate the feasibility of both available workflows. Daily MR-guided adaptive SRT of mean 25.1 min per fraction was well tolerated in this pretreated collective, and we report low rates of acute toxicity for this treatment. This study suggests that SRT on a 1.5 T MRL can be performed in clinical routine and it serves as a benchmark for future analyses.

## 1. Introduction

Hybrids of a linear accelerator and a magnetic resonance scanner (MR-Linac, MRL) have recently extended the spectrum of radiotherapeutical options. This technology allows for daily MR imaging, daily online plan adaptation and live imaging during radiotherapy (RT), and promises improvements of outcome and/or toxicity rates of RT for several tumor entities [1,2,3]. Widely performed indications for RT on an MRL seem to be anatomical sites with mobile target volumes or organs at risk (OAR) where both MR imaging and daily adaptation could help to improve current treatment outcomes. Therefore, this technology has already been evaluated for RT of primary prostate cancer, and the feasibility, safety and acute toxicity have been reported [4,5]. However, for postoperative RT (SRT) of the prostate bed in case of biochemical recurrence after radical prostatectomy (RP), there exist no clinical data on these endpoints. SRT target volumes are based on anatomical boundaries that can also be defined on (cone-beam) computed tomography (CT) [6]. Yet the improved soft-tissue contrast of MRI, in combination with the option to adapt for daily anatomical changes of OAR such as rectum and bladder, seems promising in regard to better OAR sparing, dose escalation and/or implementation of hypofractionation [7]. In this study, we examined the feasibility and acute toxicity of prospectively enrolled patients who underwent normofractionated RT of the prostate bed on a 1.5T MRL.

## 2. Methods

### 2.1. Patients

Patients were prospectively enrolled in the MRL-01 study (ClinicalTrials.gov Identifier: NCT04172753) at our department, which was approved by the institutional review board (IRB 659/2017BO1). Eligible were patients with biochemical recurrence after RP of histologically confirmed prostate cancer with cN0 or pN0 status. The indication for SRT was confirmed in an interdisciplinary tumor board. Additionally, all patients were willing to undergo consecutive native MRI scans and had no contraindications to MRI such as metal implants, pacemakers, severe claustrophobia or tinnitus.

### 2.2. Radiotherapy

Treatment consisted of 66 Gy in 33 fractions to the prostate bed or 66.5/70 Gy (simultaneously integrated boost, SIB) in 35 fractions to the prostate bed/high risk areas (R1-region, extracapsular extension) as recommended by international guidelines [8,9]. One patient was treated with 66.5/70/73.4 (SIB) Gy due to a macroscopic recurrence. Androgen deprivation therapy (ADT) was additionally given according to national guidelines [9]. A planning CT (Big Bore RT, Philips, Amsterdam, The Netherlands; 3 mm slice thickness) and a T2-weighted planning MRI on the 1.5 T MRL (Unity, Elekta, Stockholm, Sweden; 2 mm slice thickness, isotropic) were performed, fused and together served for initial contouring of clinical target volume (CTV) and OARs following the GFRU and ESTRO ACROP guidelines [6,10]. Additionally, pelvic bones, os sacrum and femora were contoured. Treatment position was supine with a moderate bladder filling protocol, and patients were instructed to empty their bowel prior to every RT session. Planning target volume (PTV) margins ranged from 6–10 mm, dorsally from 5–8 mm as was institutional routine for image-guided RT. RT was performed as step-and-shoot intensity modulated RT (IMRT) with 7MV photons using nine beam angles. Daily plan adaptation and recontouring was performed based on a native T2-weighted sequence as described in Table 1 and [11]. Planning software was Monaco v.5.40 (Elekta AB, Stockholm, Sweden). Two forms of adaptation are possible on the 1.5 T MRL Unity: “Adapt to shape” (ATS, using contour adaptation of target volumes and OARs and reoptimization of the treatment plan) or “adapt to position” (ATP, virtual couch shift without contour adaptation) [3]. The daily adaptation method was chosen by the attending radiation oncologist (DW, SB, CG) according to the presented patient anatomy of the day in comparison to the reference treatment plan and/or prior adapt-to-shape plans for each patient. New daily optimized treatment plans were verified prior to treatment using an in-house developed independent monte-carlo based secondary dose calculation [12]. 2D live imaging (balanced fast fieldecho sequence) during RT was performed in all cases to verify treatment accuracy.

### 2.3. Study Analysis

Duration of each step of the daily adaptation process was noted as well as technical or patient-sided treatment failures. Patient comfort was evaluated with validated weekly questionnaires [13]. Acute toxicity (RTOG, CTC version 4.0) was scored before RT start, weekly during RT and 3 months post RT. Additionally, patient-reported questionnaires including IPS score, ICIQ score and NCI PRO-CTCAE were evaluated prior to, during and post RT. Statistical analysis was performed using Excel 2019.

## 3. Results

### 3.1. Patient Characteristics

We enrolled 16 consecutive patients in this analysis. No patient had prior RT or severe concurring diseases. Patient characteristics are given in Table 2. RT treatment and toxicity scoring were performed as planned for all patients. Mean age at RT start was 66.4 years and mean PSA prior to RT start was 0.43 ng/mL. One patient had a macroscopic tumor recurrence in the prostatic bed as diagnosed by PSMA-PET-CT and diagnostic MRI.

### 3.2. Feasibility and Treatment Specifications

**Figure 1:**
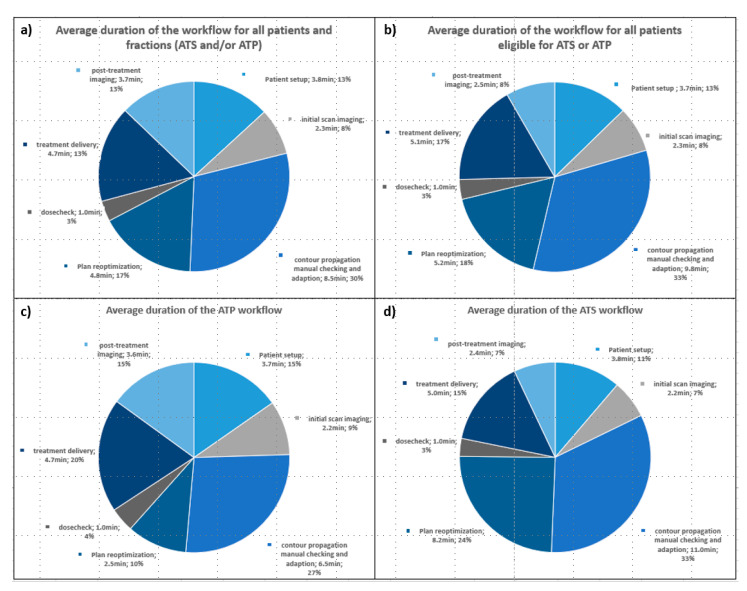
Diagrams of the consecutive substeps of the 1.5 T MR-Linac Unity workflow with average duration per substep. (**a**) All patients 1–16, including patients 1–4 who received only ATP. (**b**) Subgroup of patients 5–16, who were eligible for ATP or ATS workflow according to the attending radiation oncologist. (**c**) All ATP fractions of all patients. (**d**) All ATS fractions of all patients. ATP = adapt to shape, rigid virtual couch shift and reoptimization, ATS = adapt to shape, recontouring and optimization.

We successfully delivered 524 out of 554 treatment fractions (95.6%) on the MR-Linac. For 30 fractions, RT was performed on a conventional linac due to maintenance or unexpected technical reasons. No patient-related treatment failures occurred. Initially, in September 2018 the institution opted for ATP treatments only for all tumor sites as very limited experience was available globally and additional workflow complexity was implemented in a stepwise approach to ensure treatment safety. ATS was routinely adopted in July 2019. Therefore, the first four patients were treated exclusively with the ATP workflow and starting with patient five, ATP or ATS was performed according to the attending RO’s decision. For patients 5–16, ATP/ATS was performed in 45.8%/54.2% of fractions. In eight fractions, ATP was performed on top of the ATS workflow due to technical reasons (*n* = 3) or patient-sided reasons (patient movement, shifted anatomy) (*n* = 5). Mean duration of one treatment session (on-table time until end of RT) was 25.1 (17.6–44.8) minutes. Table 3 and Figure 1 show timings of the treatment process of various subgroups and of the consecutive substeps of the workflow.

**Figure 2:**
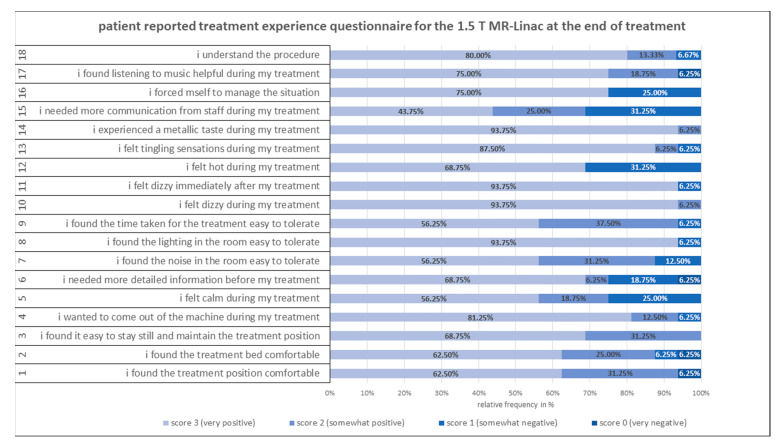
Validated patient-reported treatment questionnaire at the end of treatment using a Likert scale 1–4 for each of the 18 questions (left). Negative questions were post-processed for better comparability.

Weekly patient comfort questionnaires were completed in 99% of cases. Figure 2 shows the results of the treatment questionnaires at the end of treatment. Patients reported overall low rates of discomfort inside the MR-Linac during treatment. The most negative reports regard the need for (more) communication with the staff while inside the bore, more information about the treatment and a moderate feeling of heat.

### 3.3. Acute Toxicity

#### 3.3.1. Physician Scored Toxicity

**Figure 3:**
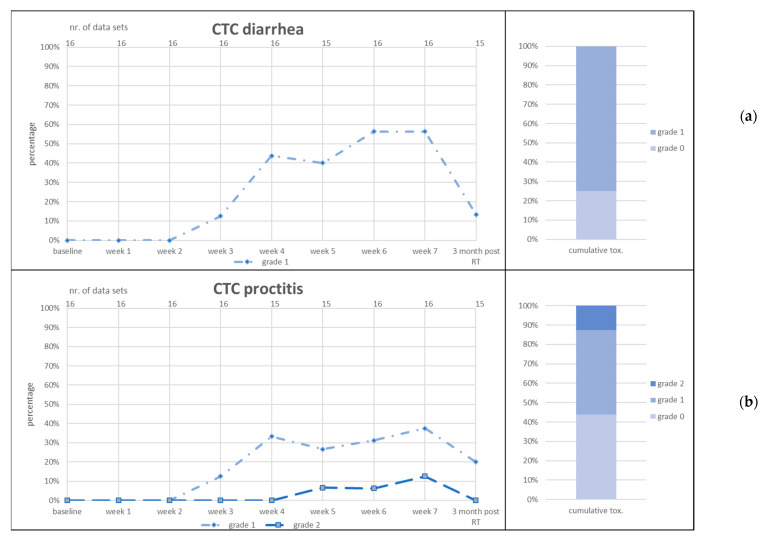
Acute gastrointestinal (GI) toxicity scored as CTC diarrhea (**a**) and CTC proctitis (**b**). RT = radiotherapy. Left graphs: number of patients in percent (*y*-axis) who reported the toxicity item at the given point of time (lower *x*-axis). Number of data sets in upper *x*-axis. Right graphs: cumulative toxicity of the given item up to three months post RT.

**Figure 4:**
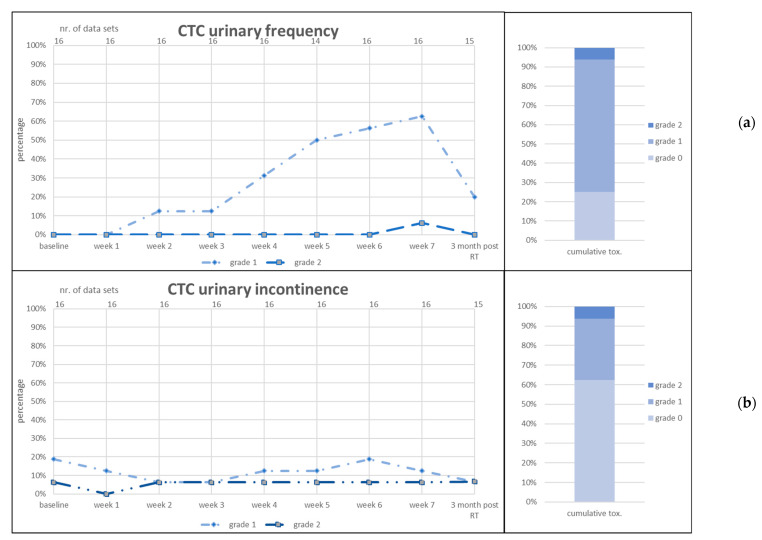
Acute genitourinary (GU) toxicity scored as CTC urinary frequency (**a**) and CTC urinary incontinence (**b**). RT = radiotherapy. Left graphs: number of patients in percent (*y*-axis) who reported the toxicity item at the given point of time (lower *x*-axis). Number of data sets in upper *x*-axis. Right graphs: cumulative toxicity of the given item up to 3 months post RT.

**Figure 5:**
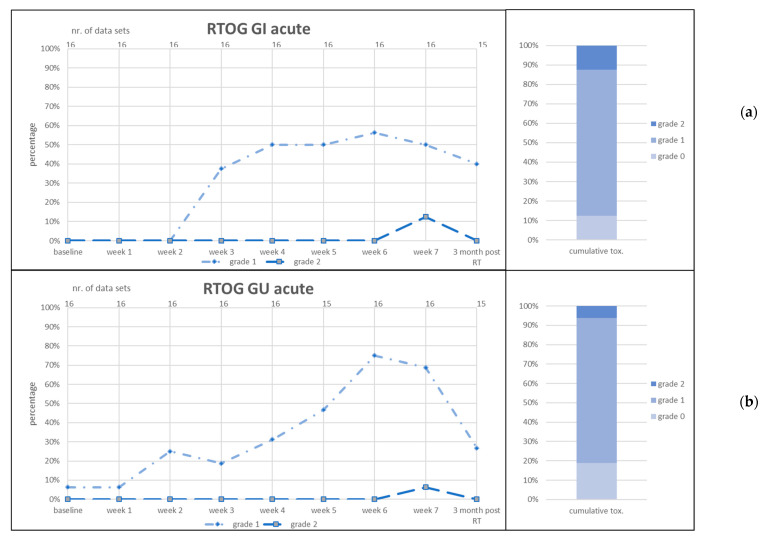
Acute gastrointestinal (GI) toxicity scored as RTOG GI acute (**a**) and genitourinary (GU) toxicity scored as RTOG GU acute (**b**). RT = radiotherapy. Left graphs: number of patients in percent (*y*-axis) who reported the toxicity item at the given point of time (lower *x*-axis). Number of data sets in upper *x*-axis. Right graphs: cumulative toxicity of the given item up to 3 months post RT.

CTC items diarrhea, proctitis, urinary frequency and urinary incontinence and RTOG GI and GU acute toxicity are given in Figure 3, Figure 4 and Figure 5.

CTC urinary urgency G1 was present in 25% (*n* = 4) of patients at baseline and increased to 40% over the course of RT. Three months post RT, 27% of patients presented G1 toxicity. CTC urinary incontinence was present at baseline in 19% (*n* = 3) of patients for G1 and in one patient of G2. These percentages did not increase during RT and decreased 3 months post RT. CTC fecal incontinence was scored for one patient at weeks 6 and 7 of RT and subsided 3 months post RT. CTC rectal bleeding was similarly scored by one patient at weeks 6 and 7 of RT as well as 3 months post RT. This patient used anticoagulants due to a concurrent cardiovascular disease. No G3+ toxicity was scored for any item.

#### 3.3.2. Patient-Reported Toxicity

Patient-reported Pro-CTCAE, ordinally scaled from 1–5, are given in the Supplementary Figures S1–S5. Scored items were genitourinary (GU) pain, GU urgency, GU frequency, GU incontinence, gastrointestinal (GI) appetite, GI abdominal pain, GI diarrhea, GI fecal incontinence, insomnia and fatigue. Mainly very mild and mild-/moderate bother was reported with a tendency towards normalization to baseline levels at 3 months post RT. No “severe bother” was scored for any item.

IPS-Score demonstrated 60%/33%/7% of mild, moderate and severe bother prior to RT (*n* = 14) compared to 38%/50%/12% at the end of RT (*n* = 14) and 80%/20%/0% (*n* = 10) 3 months post RT (Supplementary Figure S6).

The ICIQ-Score at baseline showed for 35.7% of patients (*n* = 5) no symptoms, for 21.4% (*n* = 3) mild symptoms, for 28.6% (*n* = 4) moderate symptoms and for 14.3% (*n* = 2) severe symptoms. Three months post RT, 33.3% (*n* = 3) of patients reported no symptoms, 44.4% (*n* = 4) reported mild symptoms and one patient each reported moderate and severe symptoms, respectively (Supplementary Figure S6). Patient-reported quality of life and overall health status (ordinal scale 0–7 each) worsened at the end of treatment compared to the baseline and improved again 3 months post RT to ca. 70% of scores of “excellent” health (scores 6 and 7, Supplementary Figure S6).

## 4. Discussion

This prospective study represents the first report of initial experience, feasibility and acute toxicity of MR-guided (MRg) daily adaptive SRT of prostate cancer patients on a 1.5T MRL.

### 4.1. Feasibility

We report 94.6% of fractions that could successfully be treated on the MRL. Noteworthy, the MRL in Tübingen was the first clinical setup of the Unity and initially suffered from several minor technical issues, and consequently in this phase machine failures occurred more often. Thereafter, these “teething troubles” were fixed and the MRL functioned with significantly more stability, comparable to conventional C-arm linacs. The mean treatment time of 25.1 min per fraction and the substeps of the workflow compare well to the data of de Muinck Keizer et al., who reported a mean of 33.1 min for hypofractionated primary RT of prostate cancer [14].

Our department was among the first institutions worldwide with a clinical 1.5T MRL installation. There existed no prior experience regarding the feasibility of treatment for patients with this novel technology, which was a main reason for the enrollment of all patients in a prospective study. For this same reason, we chose to perform the (easier and safer) ATP workflow for the first period to gain experience with the device. Beginning with patient five, ATP or ATS was chosen as assessed online by the attending RO. Of interest, in 54.2% of fractions, the more demanding and time consuming ATS workflow was chosen. Although the CTV for SRT is based on anatomical boundaries, in these cases the variations of bladder and/or rectal filling were deemed so extensive that the longer treatment duration and the associated burden on the patient were potentially advantageous. This highlights the potential benefit of adaptive (MR-guided) RT for this patient collective and should be considered when contemplating a reduction of PTV margins. Notably, the patient acceptance of the treatment on the MRL (on table time, noise, temperature, etc.) was very good overall and the more demanding ATS workflow is feasible in this pretreated cohort. The need for communication during and information about the treatment was one major point of criticism. This should be considered when planning postoperative RT on an MRL to increase patient compliance.

### 4.2. Acute Toxicity

We extensively report acute toxicity scored by physician as well as patient-reported outcomes, both similarly showing low toxicity without G3+ events with overall mild to moderate symptom intensity during RT, which tends to decrease again 3 months post RT. For some items, for example urinary incontinence, symptom severity was even lower than at baseline, highlighting the influence of the RP on GU toxicity. The low extent of acute toxicity of this treatment was very encouraging and is comparable to other prospective trials reporting CTC and RTOG toxicity for SRT of prostate cancer patients with normofractionated radiotherapy regimens [15,16,17]. For example, Parker et al. report early RTOG toxicity rates for 699 patients after SRT (mainly 66 Gy in 33 fractions) of diarrhea G1 + 2 of 16% (G3 < 1%), proctitis G1 + 2 of 7% (G3 < 1%) and cystitis G1 + 2 of 12% (G3 1%). Kneebone et al. found CTC toxicity rates for SRT (64 Gy in 32 fractions) of GU G1 40%, G2 42%, G3 11% and G4 1%. CTC GI toxicity was G1 35%, G2 9% and G3 1%. Noteworthy, in these recent studies, toxicity rates are given as intention-to-treat and not per-protocol percentages. This argument is further solidified by patient-reported outcomes for CTC items, IPS score and ICIQ score.

In summary, MRg SRT of prostate cancer is safe and our data support implementation into clinical routine. Of note, the more time consuming ATS workflow was subjectively assessed by the attending ROs to be advantageous in more than 50% of fractions, highlighting the advantage of this adaptive approach. Both workflows were feasible in this cohort of patients having undergone RP, which potentially causes GU side effects that limit bladder filling and therefore on-table time. We believe that the presented first results could aid other clinics in their decision of how to conduct MR SRT. Target volumes as well as PTV margins in this study did not deviate from the current GFRU guideline, which includes part of the posterior bladder [6]. Especially when using the ATS workflow, precise delineation of the bladder and bladder wall is possible on MRI, and target volumes could be adapted accordingly. However, the posterior/inferior bladder wall itself is clearly part of the CTV, and organ motion during ATS needs to be considered. We plan to expand the cohort and analyze whether an adaptation of the CTV or a reduction of PTV margins might be possible.

Whether MRg SRT—with any of the two available workflows—will result in reduced late toxicity and how this approach compares with respect to outcome parameters cannot be answered yet, for clinical MRL treatments were initiated in 2018, and outcome data of 10 years or more is necessary to validly answer this question. The present study represents a basis for further (prospective, multicentric) studies that seem necessary to answer the question of comparability of adaptive approaches on MRL- and CT-based linacs.

## 5. Limitations

The overall number of patients in this study cohort is limited (*n* = 16) yet represents a benchmark for further analyses. Additionally, the included patients are heterogenous regarding treatment doses; however, all prescribed total doses were in accordance with international recommendations. The first four consecutive patients were treated with ATP only to gain experience with this simpler workflow. In addition, patients in this study were selected by MRI eligibility: patients needed to be able and willing to undergo consecutive MRI scans. This selection bias limits the comparability of the study cohort with other studies. However, we believe that the conclusions of this study nevertheless remain valid. We mention that the MR acceptance results of *n* = 7 patients in this study were already included in the multicenter validation study of the MR acceptance questionnaire by Barnes et al. [12] and have already been published.

## 6. Conclusions

In this prospective study we were able to demonstrate the feasibility and low acute toxicity of MR-guided SRT of patients with biochemical recurrence of prostate cancer. Both workflows ATP and ATS are feasible in this pretreated population. This study serves as a first reference point for institutions that aim to implement SRT on a 1.5 T MRL.

## Figures and Tables

**Table 1:** MRI sequence parameters used for daily plan adaption.

T2 3D Tra 2 Min			
Field of view [mm] (AP × RL × FH)	400	400	300
Acquired voxel size [mm] (AP × RL × FH)	1.5	1.5	2
Reconstructed voxel size [mm] (AP × RL × FH)	0.83	0.83	1
Flip angle [°]	90		
TR [ms]	1535		
TE [ms]	278		
WFS [Pixel]/BW [Hz]	0.293/740.3		
Scantime [min]	01:57		

TR = Repetition Time, TE = Echo Time. WFS = Water Fat Shit. BW = Band Width.

**Table 2:** Characteristics of the patient collective.

Parameter	Mean (Range)	Median
Age at RT start (years, (range))	66.4 (55–77)	65.5
Interval from RP to RT start (months)	45.2 (6–120)	34
Imaging prior to RT (n, %).		
CT	1 (6.25%)	
MRI	11 (68.75%)	
PSMA-PET-CT	9 (56.25%)	
Gleason-Score (n, %)		
7a	10 (62.5%)	
7b	4 (25.0%)	
8	1 (6.25%)	
9	1 (6.25%)	
Tumor stage (n, %)		
pT2a	2 (12.5%)	
pT2c	11 (68.75%)	
pT3a	2 (12.5%)	
pT3b	1 (6.25%)	
Resection-status (n, %)		
R0	7 (43.75%)	
R1	7 (43.75%)	
R2	0 (0.0%)	
RX	2 (12.5%)	
PSA Value in ng/mL		
Prior to RT	0.43 (0.07–3.4)	0.23
3 months post RT	0.06 (<0.004–0.15)	0.06
6 months post RT	0.04 (<0.004–0.1)	0.03
Total RT dose		
66 Gy	6 (37.5%)	
70 Gy	9 (56.25%)	
73.5 Gy	1 (6.25%)	
Additional ADT (n, %)	6 (37.5%)	

RT = radiotherapy. RP = radical prostatectomy. CT = computer tomography. MRI = magnetic resonance imaging. PSMA-PET-CT = prostate-specific membrane antigen-position emission tomography CT. PSA = prostate specific antigen. ADT = androgen deprivation therapy additional to RT of 6–36 months.

**Table 3:** Duration of the treatment process by subgroup.

Parameter	Mean (Range) in Minutes	Median in Minutes
**all patients (nrs. 1–16)**		
duration start to post-imaging	25.1 (17.6–44.8)	24.7
duration start to RT	20.3 (14.4–40.4)	19.0
**subgroup ATP&ATS (nrs. 5–16)**		
duration start to post-imaging	27.1 (17.6–44.8)	26.7
duration start to RT	22.0 (14–40.4)	21.7
**ATP fractions only**		
duration start to post-imaging	20.6 (17.6–25.2)	20.6
duration start to RT	15.9 (14–20.8)	15.5
**ATS fractions only**		
duration start to post-imaging	31.3 (28.2–34.1)	31.4
duration start to RT	26.3 (23.4–29.7)	26.2

RT = Radiotherapy. ATP = adapt to position, “rigid” workflow with virtual couch shift. ATS = adapt to shape, recontouring and replanning.

## Data Availability

The datasets used and/or analyzed during the current study are available from the corresponding author on reasonable request.

## Supplementary Materials

The following supporting information can be downloaded at: https://www.mdpi.com/article/10.3390/jcm11164651/s1, Figure S1: Patient-reported outcomes (pro) of acute genitourinary (GU) toxicity scored as CTC GU pain (**a**) and CTC GU urgency (**b**). RT = radiotherapy. Left graphs: number of patients in percent (y-axis) who reported the toxicity item at the given point of time (x-axis). Right graphs: cumulative toxicity of the given item up to 3 months post RT.; Figure S2: Patient-reported outcomes (pro) of acute genitourinary (GU) toxicity scored as CTC GU frequency (**a**) and CTC GU incontinence (**b**). RT = radiotherapy. Left graphs: number of patients in percent (*y*-axis) who reported the toxicity item at the given point of time (*x*-axis). Right graphs: cumulative toxicity of the given item up to 3 months post RT.; Figure S3: Patient-reported outcomes (pro) of acute gastrointestinal (GI) toxicity scored as CTC GI appetite (**a**) and CTC GI abdominal pain (**b**). RT = radiotherapy. Left graphs: number of patients in percent (*y*-axis) who reported the toxicity item at the given point of time (*x*-axis). Right graphs: cumulative toxicity of the given item up to 3 months post RT.; Figure S4: Patient-reported outcomes (pro) of acute gastrointestinal (GI) toxicity scored as CTC GI diarrhea (**a**) and CTC GI fecal incontinence (**b**). RT = radiotherapy. Left graphs: number of patients in percent (*y*-axis) who reported the toxicity item at the given point of time (*x*-axis). Right graphs: cumulative toxicity of the given item up to 3 months post RT.; Figure S5: Patient-reported outcomes (pro) of acute toxicity scored as CTC insomnia (**a**) and CTC fatigue (**b**). RT = radiotherapy. Left graphs: number of patients in percent (y-axis) who reported the toxicity item at the given point of time (x-axis). Right graphs: cumulative toxicity of the given item up to 3 months post RT.; Figure S6: Patient-reported outcome of ICIQ score (**a**), IPS score (**b**), quality of life (**c**) and overall health status (**d**). (**c**,**d**) are scored on an ordinal scale from 0 (worst) to 7 (optimal).
