# Supplementary material for: First Experience and Prospective Evaluation on Feasibility and Acute Toxicity of Online Adaptive Radiotherapy of the Prostate Bed as Salvage Treatment in Patients with Biochemically Recurrent Prostate Cancer on a 1.5T MR-Linac
Source: J Clin Med. 2022 Aug 9;11(16):4651. doi: 10.3390/jcm11164651 (PMC9410121; doi:10.3390/jcm11164651)
Supplement: Supplementary file 1 [file jcm-11-04651-s001.zip › jcm-1798951-supplementary.pdf]

Supplementary Figures:

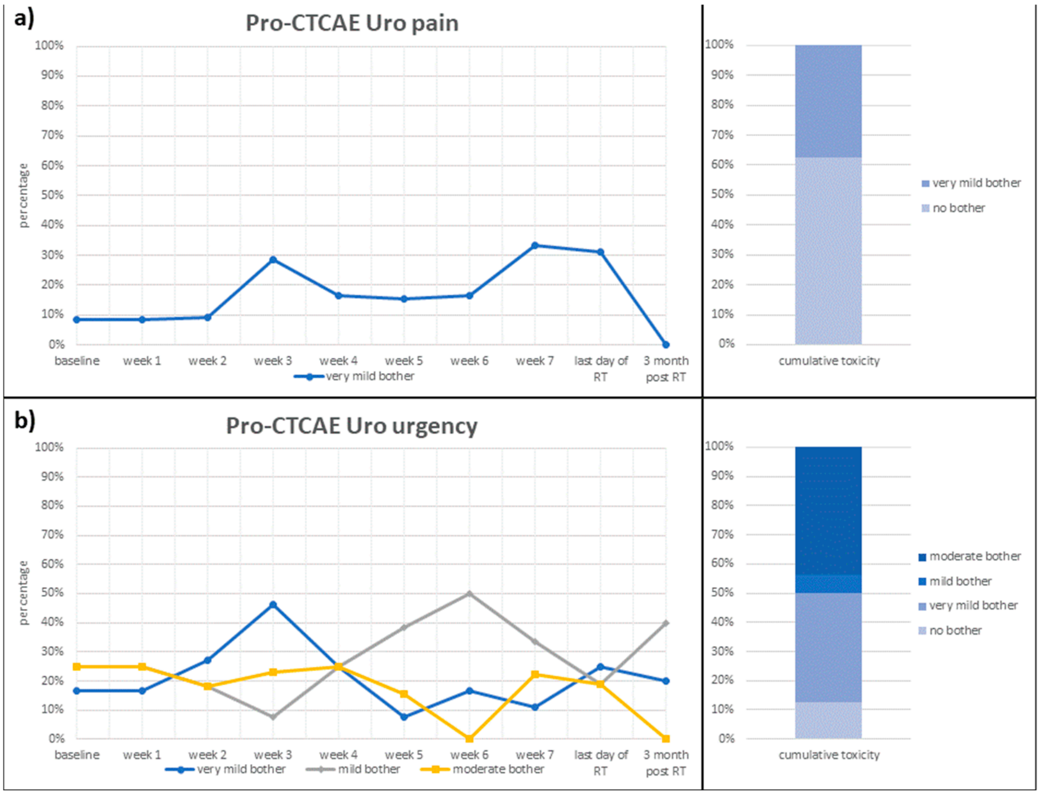

Figure S1. Patient reported outcomes (pro) of acute genitourinary (GU) toxicity scored as CTC GU pain (a) and CTC GU urgency (b). RT = radiotherapy. Left graphs: number of patients in percent (y-axis) who reported the toxicity item at the given point of time (x-axis). Right graphs: cumulative toxicity of the given item up to three months post RT.

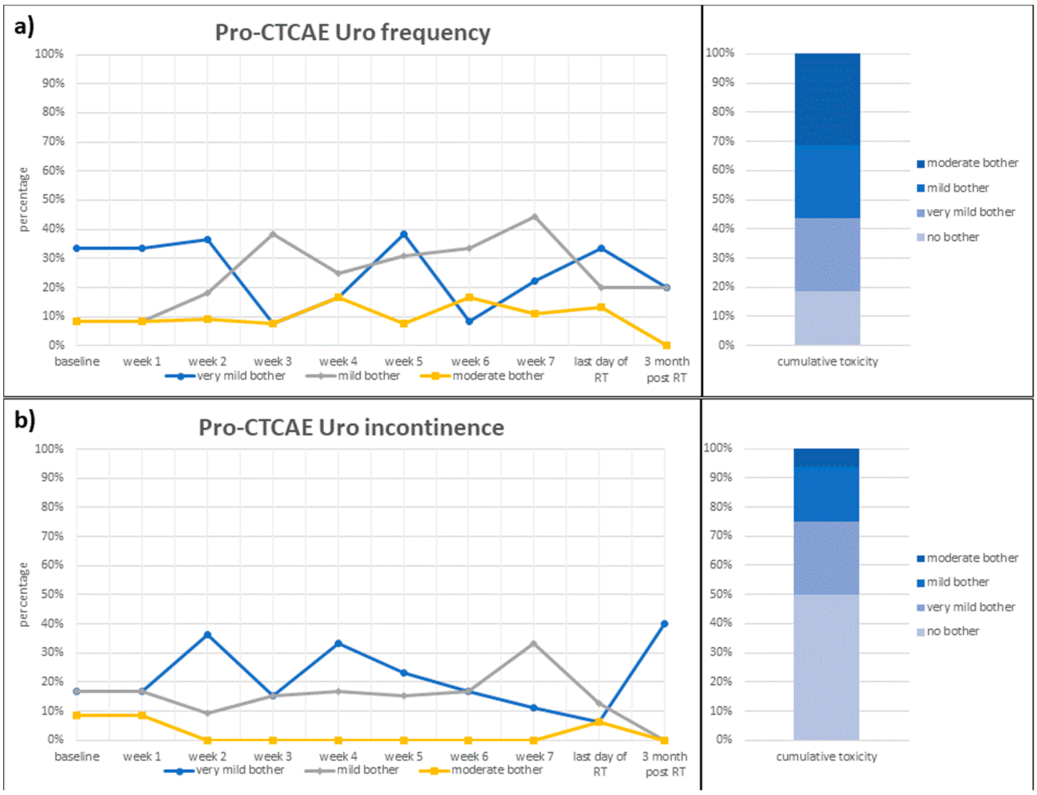

Figure S**2.** Patient reported outcomes (pro) of acute genitourinary (GU) toxicity scored as CTC GU frequency (a) and CTC GU incontinence (b). RT = radiotherapy. Left graphs: number of patients in percent (y-axis) who reported the toxicity item at the given point of time (x-axis). Right graphs: cumulative toxicity of the given item up to three months post RT.

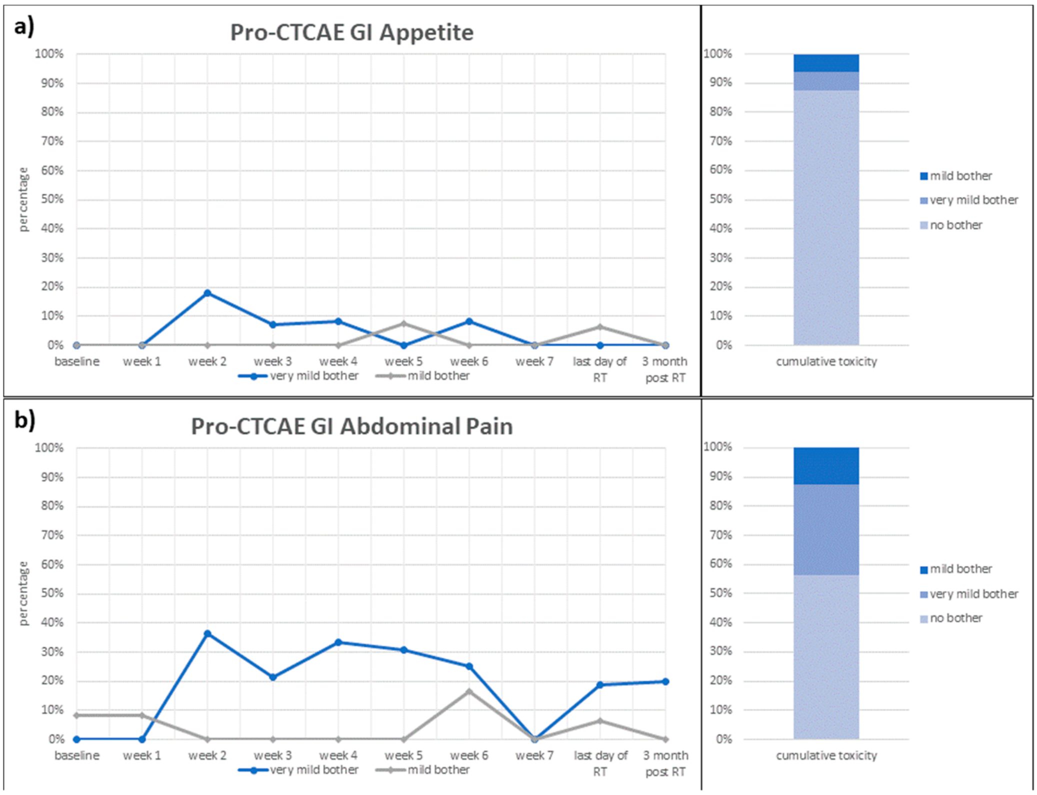

Figure S**3.** Patient reported outcomes (pro) of acute gastrointestinal (GI) toxicity scored as CTC GI appetite (a) and CTC GI abdominal pain (b). RT = radiotherapy. Left graphs: number of patients in percent (y-axis) who reported the toxicity item at the given point of time (x-axis). Right graphs: cumulative toxicity of the given item up to three months post RT.

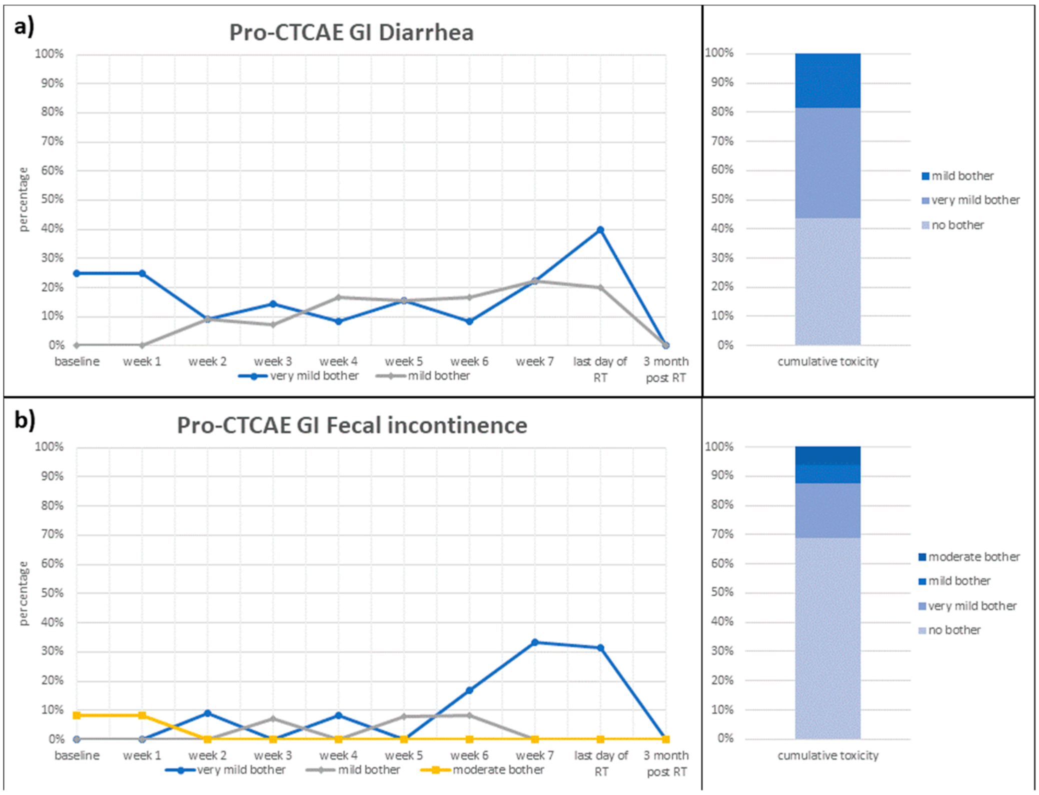

Figure S4. Patient reported outcomes (pro) of acute gastrointestinal (GI) toxicity scored as CTC GI diarrhea (a) and CTC GI fecal incontinence (b). RT = radiotherapy. Left graphs: number of patients in percent (y-axis) who reported the toxicity item at the given point of time (x-axis). Right graphs: cumulative toxicity of the given item up to three months post RT.

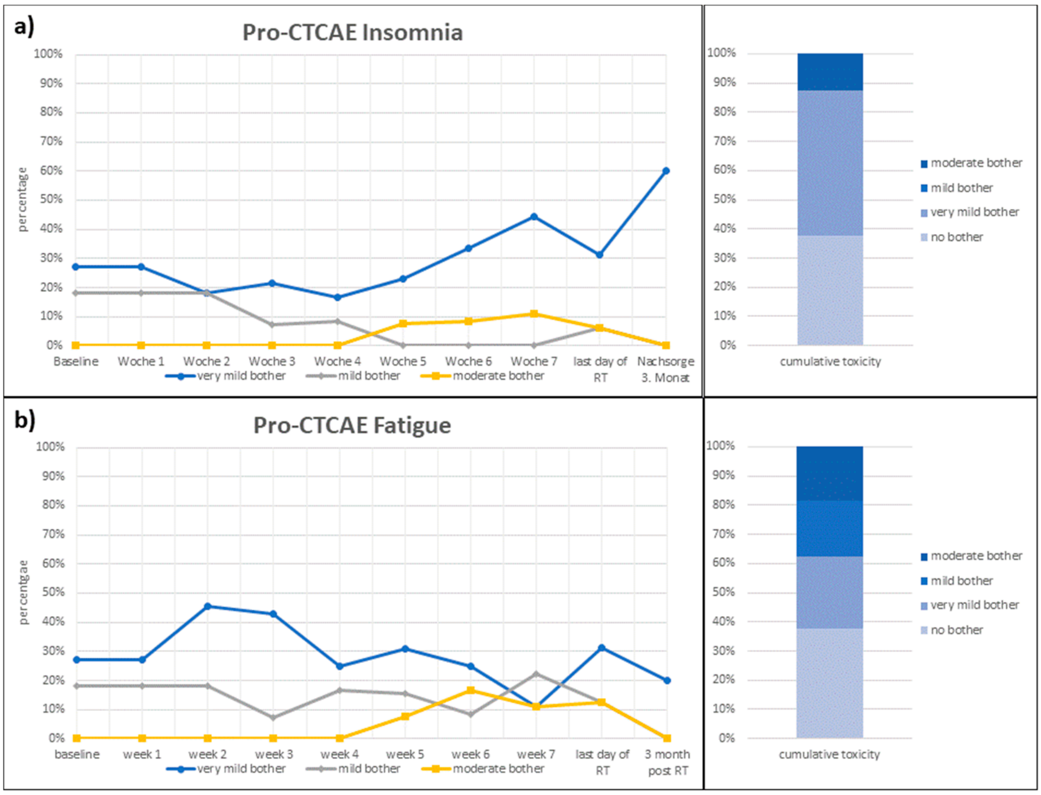

Figure S**5. P**atient reported outcomes (pro) of acute toxicity scored as CTC insomnia (a) and CTC fatigue (b). RT = radiotherapy. Left graphs: number of patients in percent (y-axis) who reported the toxicity item at the given point of time (x-axis). Right graphs: cumulative toxicity of the given item up to three months post RT.

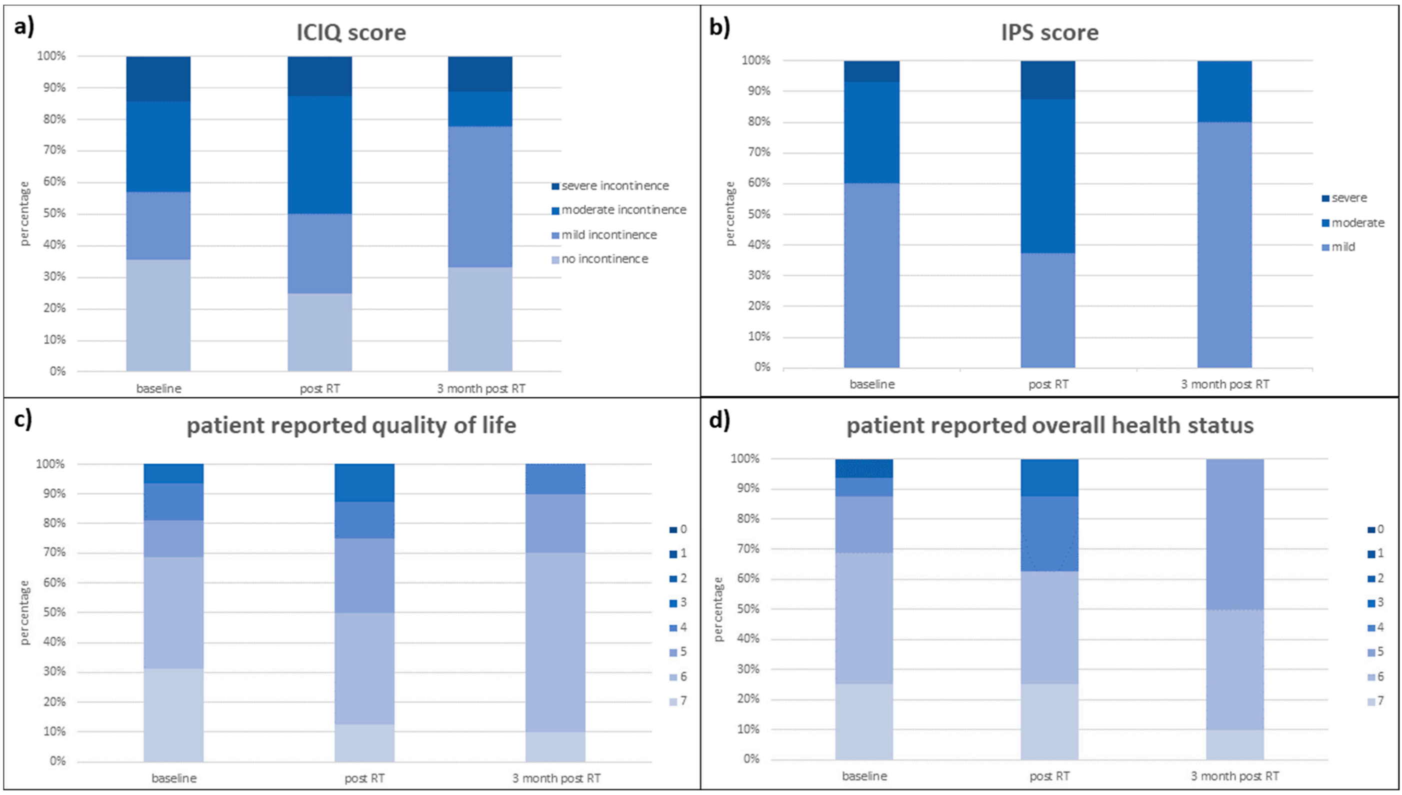

Figure S**6.** Patient reported outcome of ICIQ score (a), IPS score (b), quality of life (c) and overall health status (d). (c) and (d) are scored on an ordinal scale from 0 (worst) to 7 (optimal).
